# Supplementary material for: Management of Obesity During Pregnancy and Periconception: Case-Based Learning for OB/GYN Clerkships
Source: MedEdPORTAL. 2021 Mar 23;17:11129. doi: 10.15766/mep_2374-8265.11129 (PMC8015635; doi:10.15766/mep_2374-8265.11129)
Supplement: Supplementary file 1 — Project Implicit Introduction.docxAdvance Preparation Student Version.docxFacilitator Guide.docxPreseminar Quiz Student Version.docxDiscussion Questions Student Version.docxPostseminar Feedback Survey.docx [file mep_2374-8265.11129-s001.zip › A. Project Implicit Introduction.docx]

**PROJECT IMPLICIT ® INTRODUCTION**

***The following excerpt was obtained from the Project Implicit® website and is included as Appendix A for your reading convenience:***

“The IAT measure the strength of associations between concepts (e.g., black people, gay people) and evaluations (e.g., good, bad) or stereotypes (e.g., athletic, clumsy). The main idea is that making a response is easier when closely related items share the same response key.

When doing an IAT you are asked to quickly sort words into categories that are on the left and right hand side of the computer screen by pressing the “e” key if the word belongs to the category on the left and the “I” key if the word belongs to the category on the right. The IAT has five main parts.

In the first part of the IAT you sort words relating to the concepts (e.g., fat people, thin people) into categories. So if the category “Fat People” was on the left, and a picture of a heavy person appeared on the screen, you would press the “e” key.

In the second part of the IAT you sort words relating to the evaluation (e.g., good, bad). So if the category “good” was on the left, and a pleasant word appeared on the screen, you would press the “e” key.

In the third part of the IAT the categories are combined and you are asked to sort both concept and evaluation words. So the categories on the left hand side would be Fat People/Good and the categories on the right hand side would be Thin People/Bad. It is important to note that the order in which the blocks are presented varies across participants, so some people will do the Fat People/Good, Thin People/Bad part first and other people will do the Fat People/Bad, Thin People/Good part first.

In the fourth part of the IAT the placement of the concepts switches. If the category “Fat People” was previously on the left, now it would be on the right. Importantly, the number of trials in the part of the IAT is increased in order to minimize the effects of practice.

In the final part of the IAT the categories are combined in a way that is opposite what they were before. If the category on the left was previously Fat People/Good, it would now be Fat People/Bad.

The IAT score is based on how long it takes a person, on average, to sort the words in the third part of the IAT versus the fifth part of the IAT. We would say that one has an implicit preference for thin people relative to fat people if they are faster to categorize words when Thin People and Good share a response key and Fat People and Bad share a response key, relative to the reverse.” ^1^

**Reference:**

1. Ratliff K, Bar-Anan Y, Lai C, Smith CT, Nosek B, Greenwald T. Project Implicit. 2011. https://implicit.harvard.edu/implicit/iatdetails.html. Published 2020.
