# Supplementary material for: Management of Obesity During Pregnancy and Periconception: Case-Based Learning for OB/GYN Clerkships
Source: MedEdPORTAL. 2021 Mar 23;17:11129. doi: 10.15766/mep_2374-8265.11129 (PMC8015635; doi:10.15766/mep_2374-8265.11129)
Supplement: Supplementary file 1 — Project Implicit Introduction.docxAdvance Preparation Student Version.docxFacilitator Guide.docxPreseminar Quiz Student Version.docxDiscussion Questions Student Version.docxPostseminar Feedback Survey.docx [file mep_2374-8265.11129-s001.zip › B. Advance Preparation Student Version.docx]

**Management of Obesity During Pregnancy and Periconception: Case-Based Learning for OB/GYN Clerkships**

*Authors:* James Cook, MD,^1^ Hannah L. Puckett, BS,^2^ Jody E. Steinauer, MD, MAS^3^

*Affiliations:* ^1^Department of OB/GYN, Prisma Health Midlands Affiliate, University of South Carolina School of Medicine Columbia*;* ^2^MD Candidate, University of South Carolina School of Medicine Columbia*;* ^3^Department of Obstetrics, Gynecology, and Reproductive Sciences, Division of Zuckerberg San Francisco General, University of California – San Francisco

*Key* *Words:*  Bariatric Surgery, Obesity, Pregnancy, Reproductive Health

**Evidence-Based Education Project:**

*Problem:*There is lack of knowledge among medical students about the effects of bariatric surgery on pregnancy and the medical management of obesity as it relates to reproductive health.  Additionally, there is bias toward obese patients among clinicians and learners.

*Intervention:*We designed a flipped classroom learning experience focused on teaching medical students about the impact and management of obesity and bariatric surgery on pregnancy and reproductive health, with the use of pre-reading and clinical vignettes.  Students took the Implicit Attitude Test (IAT) before the seminar and read two review articles: an ACOG Practice Bulletin and Endocrine Society Practice guideline.  During a 60-minute, in-person seminar students first worked in small groups to discuss clinical vignettes and answer questions/design treatment plans (45 minutes), and then discussed their results on the IAT and how bias can affect patient care.  Faculty preceptors oversaw the work and led the discussion.

*Context:*This module was designed for and implemented into the OB/GYN clerkship curriculum for third-year medical students, but it could also be used for fourth-year medical students or resident learners. It was piloted to a total of 20 students between December 2018 and March 2019.

*Outcome/Lessons Learned:*We measured basic knowledge about obesity and bariatric surgery (using pre- and post-seminar quizzes, with facts taken from the pre-reading and learning objectives), and their impacts on pregnancy and reproductive health. We also assessed students’ feelings about the seminar's discussion regarding bias toward obese patients during the post-seminar feedback survey.

**Educational Objectives:**

By the end of this session, learners will be able to:

1. Explain the maternal and fetal effects of obesity on pregnancy.
2. Identify the indications for using pharmacotherapy versus bariatric surgery to manage obesity in a patient who desires future fertility.
3. Discuss the effects bariatric surgery has on future fertility and contraception.
4. Understand the impact bariatric surgery can have on future pregnancies.
5. Reflect on how implicit bias impacts the delivery of patient care to obese patients.

**ADVANCED PREPARATION STUDENT INSTRUCTIONS**

**Before the seminar:**

1. Read the Project Implicit® excerpt included in Appendix A.
2. Then proceed with the Implicit Attitude Test (IAT): <https://implicit.harvard.edu/implicit/>
   1. No need to log on, continue as a guest and click “I wish to proceed” 🡪 select weight IAT and take the test
3. Read the following review articles:
   1. Kominiarek MA. ACOG Practice Bulletin Clinical Management Guidelines for Obstetrician – Gynecologists: Bariatric Surgery and Pregnancy. *Am Coll Obstet Gynecol*. 2019;114(106):192-202. doi:10.1097/AOG.0b013e318188d1c2
   2. Apovian CM, Aronne LJ, Bessesen DH, et al. Pharmacological management of obesity: An endocrine society clinical practice guideline. *J Clin Endocrinol Metab*. 2015;100(2):342-362. doi:10.1210/jc.2014-3415
