# Supplementary material for: Management of Obesity During Pregnancy and Periconception: Case-Based Learning for OB/GYN Clerkships
Source: MedEdPORTAL. 2021 Mar 23;17:11129. doi: 10.15766/mep_2374-8265.11129 (PMC8015635; doi:10.15766/mep_2374-8265.11129)
Supplement: Supplementary file 1 — Project Implicit Introduction.docxAdvance Preparation Student Version.docxFacilitator Guide.docxPreseminar Quiz Student Version.docxDiscussion Questions Student Version.docxPostseminar Feedback Survey.docx [file mep_2374-8265.11129-s001.zip › C. Facilitator Guide.docx]

**Management of Obesity During Pregnancy and Periconception: Case-Based Learning for OB/GYN Clerkships**

*Authors:* James Cook, MD,^1^ Hannah L. Puckett, BS,^2^ Jody E. Steinauer, MD, MAS^3^

*Affiliations:* ^1^Department of OB/GYN, Prisma Health Midlands Affiliate, University of South Carolina School of Medicine Columbia*;* ^2^MD Candidate, University of South Carolina School of Medicine Columbia*;* ^3^Department of Obstetrics, Gynecology, and Reproductive Sciences, Division of Zuckerberg San Francisco General, University of California – San Francisco

*Key* *Words:*  Bariatric Surgery, Obesity, Pregnancy, Reproductive Health

**Contents:**

Facilitator’s Guide: Before the Seminar

Post-Seminar Quiz without Answers – *To be printed for each student participant before the start of the seminar*

Post-Seminar Feedback Survey – *To be printed for each student participant before the start of the seminar*

Pre-Seminar Quiz with Answers

Facilitator’s Guide: During the Seminar

Discussion Questions with Answers

Post-Seminar Quiz with Answers

**Evidence-Based Education Project:**

*Problem:*There is lack of knowledge among medical students about the effects of bariatric surgery on pregnancy and the medical management of obesity as it relates to reproductive health.  Additionally, there is bias toward obese patients among clinicians and learners.

*Intervention:*We designed a flipped classroom learning experience focused on teaching medical students about the impact and management of obesity and bariatric surgery on pregnancy and reproductive health, with the use of pre-reading and clinical vignettes.  Students took the Implicit Attitude Test (IAT) before the seminar and read two review articles: an ACOG Practice Bulletin and Endocrine Society Practice guideline.  During a 60-minute, in-person seminar students first worked in small groups to discuss clinical vignettes and answer questions/design treatment plans (45 minutes), and then discussed their results on the IAT and how bias can affect patient care.  Faculty preceptors oversaw the work and led the discussion.

*Context:*This module was designed for and implemented into the OB/GYN clerkship curriculum for third-year medical students, but it could also be used for fourth-year medical students or resident learners. It was piloted to a total of 20 students between December 2018 and March 2019.

*Outcome/Lessons Learned:*We measured basic knowledge about obesity and bariatric surgery (using pre- and post-seminar quizzes, with facts taken from the pre-reading and learning objectives), and their impacts on pregnancy and reproductive health. We also assessed students’ feelings about the seminar's discussion regarding bias toward obese patients during the post-seminar feedback survey.

**Educational Objectives:**

By the end of this session, learners will be able to:

1. Explain the maternal and fetal effects of obesity on pregnancy.
2. Identify the indications for using pharmacotherapy versus bariatric surgery to manage obesity in a patient who desires future fertility.
3. Discuss the effects bariatric surgery has on future fertility and contraception.
4. Understand the impact bariatric surgery can have on future pregnancies.
5. Reflect on how implicit bias impacts the delivery of patient care to obese patients.

**FACILITATOR’S GUIDE: *BEFORE THE SEMINAR***

1. Facilitator should advise students to prepare ahead of time for the seminar with the following instructions (also included in the Student Version of the handout):
   1. Read the Project Implicit® excerpt included in Appendix A.
   2. Then proceed with the Implicit Attitude Test (IAT): <https://implicit.harvard.edu/implicit/>
      1. No need to log on, continue as a guest and click “I wish to proceed” 🡪 select weight IAT and take the test
   3. Read the following review articles:
      1. Kominiarek MA. ACOG Practice Bulletin Clinical Management Guidelines for Obstetrician – Gynecologists: Bariatric Surgery and Pregnancy. *Am Coll Obstet Gynecol*. 2019;114(106):192-202. doi:10.1097/AOG.0b013e318188d1c2
      2. Apovian CM, Aronne LJ, Bessesen DH, et al. Pharmacological management of obesity: An endocrine society clinical practice guideline. *J Clin Endocrinol Metab*. 2015;100(2):342-362. doi:10.1210/jc.2014-3415
2. Prior to the start of the seminar, the facilitator should print out enough post-seminar quizzes and feedback surveys *(located on the following three pages)* for each student participant.

*********PRIOR TO THE SEMINAR, PRINT THE FOLLOWING THREE PAGES FOR EACH STUDENT PARTICIPANT*********

**OBESITY CBL: POST-SEMINAR QUIZ**

**CASE #1: Patient is a 25-year-old G0 with Type 2 Diabetes Mellitus, Chronic Hypertension and BMI 48 presenting for preconception counseling. She is currently taking metformin and propranolol.**

1. What are some of the effects of obesity on this patient’s fertility?

a. Obesity can cause anovulation and oligo-ovulation.

b. Obese patients are more likely to respond to ovulation induction when given higher doses of gonadotropins.

c. Due to changes in estrogen levels, fertility actually increases in obese patients.

d. Obese patient have the same risk of miscarriage as the general population.

2. Which of the following is a suitable medication change for this patient considering that she is trying to become pregnant?

a. She should add orlistat in order to help her lose weight.

b. In addition to continuing her metformin, she should add a sulfonylurea to her medications in order to better control her diabetes.

c. She should switch from propranolol to lisinopril due to its renal protection in diabetes.

d. She should add liraglutide to her medications due to its weight loss properties.

3. Which of the following patients meet criteria for bariatric surgery?

a. 32 yo G1P1 with a BMI of 32 with type II diabetes

b. 28 yo G2P2 with a BMI of 38 who is otherwise healthy

c. 40 yo G3P2 with a BMI of 41 with sleep apnea

d. 25 yo G0P0 with a BMI of 34 with hypertension

**CASE #2: Patient is a 29-year-old G2P2002 status post gastric banding who previously had a BMI of 31 presenting for annual exam. She is also interested in contraception.**

4. Why is contraceptive counseling important in this patient?

a. Pregnancy rates after bariatric surgery are the same compared to the adolescent population.

b. It is recommended that patients wait 6 months after bariatric surgery to conceive.

c. There is increased risk of oral contraception failure should the patient choose to use COCs.

d. The patient can only use a non-hormonal option, such as a copper IUD, for contraception use.

5. Of the following, which contraceptive option is best for this patient following surgery?

a. Progestin-only pills

b. Combined oral contraception

c. Nexplanon

d. Copper IUD

6. Should this patient become pregnant, which of the following is false regarding the considerations that must be made for her history of gastric banding?

a. Removing fluid from the gastric band may aid in reducing nausea/vomiting in T1.

b. She may experience decreased food intake or food intolerance.

c. Due to this being a restrictive procedure rather than a malabsorptive procedure, the patient is at less risk of nutritional deficiencies.

d. Consultation with a bariatric surgeon is recommended.

**CASE #3: Patient is a 38-year-old G3P2002 at 9w3d, with a history of Roux-en-Y procedure, presenting to initiate prenatal care.**

7. How will this patient’s pregnancy compare to her pregnancies prior to the Roux-en-Y procedure?

a. There is a decreased rate of cesarean section.

b. There is a decreased rate of gestational diabetes.

c. There is a decreased rate of preterm delivery.

d. There is a decreased rate of PPROM.

8. According to some case reports, what is a severe complication of bariatric surgery that has been noted in pregnancy?

a. Gastrointestinal hemorrhage

b. Infections

c. Fistula formations

d. Gastrointestinal ileus

9. Which of the following is not a consideration that needs to be made during this patient's pregnancy?

a. There may be a delay in bariatric-related complications.

b. All GI problems must be thoroughly evaluated.

c. Exploratory surgery during pregnancy is not recommended due to possible risks.

d. Late severe complications of surgery may occur in pregnancy.

10. The patient presents to you during her pregnancy with abdominal cramps, diarrhea, and nausea/vomiting. Given her history of bariatric surgery, what is the likely diagnosis and what prenatal screening test does this affect?

a. Bowel obstruction; glucose challenge test

b. Ileus; urinalysis

c. GI leak; urinalysis

d. Dumping syndrome; glucose challenge test

**OBESITY IN PREGNANCY CBL: POST-SEMINAR FEEDBACK SURVEY**

**Directions: Reflect on the seminar you just participated in by reading the following statements and circling the corresponding descriptive word/phrase (from “strongly agree” to “strongly disagree”) describing your level of agreement with it.**

1. Bias testing using the IAT was a meaningful experience for me.

Strongly agree Agree Neither agree nor disagree Disagree Strongly disagree

1. The discussion of cognitive bias was useful to me as a medical student.

Strongly agree Agree Neither agree nor disagree Disagree Strongly disagree

1. I will now be more intentional in considering my own bias when interacting with obese patients.

Strongly agree Agree Neither agree nor disagree Disagree Strongly disagree

1. The layout of the flipped classroom obesity CBL lesson was more engaging than a traditional CBL.

Strongly agree Agree Neither agree nor disagree Disagree Strongly disagree

1. The quizzes helped reinforce information taught in the obesity CBL.

Strongly agree Agree Neither agree nor disagree Disagree Strongly disagree

1. The CBL teaching method enabled me to be a more active learner than in the typical lecture-based style of teaching.

Strongly agree Agree Neither agree nor disagree Disagree Strongly disagree

1. Overall, I prefer the CBL method of teaching over the typical lecture-based style.

Strongly agree Agree Neither agree nor disagree Disagree Strongly disagree

1. Please leave any additional comments or concerns here:

**OBESITY CBL FACILITATOR VERSION: PRE-SEMINAR QUIZ**

**CASE #1: Patient is a 25-year-old G0 with Type 2 Diabetes Mellitus, Chronic Hypertension and BMI 48 presenting for preconception counseling. She is currently taking metformin and propranolol.**

1. Should this patient become pregnant, which of the following is false in regard to the maternal effects of obesity on her pregnancy?

a. This patient is at increased risk of gestational diabetes and preeclampsia.

b. This patient is more likely to have a spontaneous preterm labor.

c. This patient is more likely to have a cesarean delivery.

d. This patient is more likely to have a longer labor.

2. What should you tell this patient about the fetal and neonatal effects of obesity in pregnancy?

a. Being obese compromises fetal weight estimations.

b. Prenatal congenital anomalies are more likely to be diagnosed via ultrasound visualization in these patients.

c. There is a decreased risk of stillbirth in these patients.

d. There is an increased chance of birth defects relating to cardiac systems and facial clefting.

3. Should this patient undergo bariatric surgery, what effect will that have on her future fertility?

a. Rapid weight loss following surgery causes hormonal changes that decrease fertility.

b. There is the potential for compromised absorption of COCs following surgery.

c. Rapid weight loss can cause irregular menses following surgery.

d. She should wait at least 6 months after surgery to conceive so that the fetus is not exposed to rapid maternal weight loss.

**CASE #2: Patient is a 29-year-old G2P2002 status post gastric banding who previously had a BMI of 31 presenting for annual exam. She is also interested in contraception.**

4. Prior to her surgery, which of the following contraception options would be contraindicated in this patient?

a. Nexplanon

b. COCs

c. Depo shot

d. Levonorgestrel IUD

5. Which of the following is the least likely nutritional deficiency following bariatric surgery?

a. Iron

b. Folate

c. Vitamin B6

d. Calcium

6. Should this patient become pregnant; how will you manage her possible nutritional deficiencies?

a. Do a broad evaluation for micronutrient deficiencies starting in T2.

b. Get a CBC and micronutrient levels every trimester.

c. Start with parenteral supplementation if a specific nutritional deficit is noted.

d. Prescribe higher doses of folate due to her history of bariatric surgery.

**CASE #3: Patient is a 38-year-old G3P2002 at 9w3d, with a history of Roux-en-Y procedure, presenting to initiate prenatal care.**

7. What is an important consideration for this patient who has undergone bariatric surgery?

a. This patient has an increased risk of hypertension after surgery.

b. This patient is less likely to develop gestational diabetes when compared to the general public.

c. This patient has an increased risk of preeclampsia following surgery.

d. This patient is likely to still be obese, and this must be considered when interpreting studies and clinical care options.

8. Which of the following is false regarding the effect of bariatric surgery on the rates of cesarean section?

a. When compared to obese controls, there is no significant difference in rate of cesarean section.

b. When compared to the general population, bariatric patients are more likely to have had a prior cesarean section.

c. When compared to severely obese controls, there are lower rates of cesarean section delivery rates after bariatric surgery.

d. When compared to nonobese patients who have not undergone surgery, there are higher cesarean section delivery rates after bariatric surgery.

9. Which of the following is an effect of previous bariatric surgery on the fetus?

a. The fetus is at an increased risk of a congenital anomaly following bariatric surgery.

b. The rate of fetal macrosomia is the same compared to pre-bariatric surgery rates.

c. The baby is less likely to be large for gestational age compared to pre-bariatric surgery rates.

d. The baby is at an increased risk of perinatal death.

10. Which of the following is false regarding labor and delivery for this patient?

a. This patient may have a shorter labor time.

b. This patient may need labor induction.

c. This patient may be admitted earlier in labor.

d. This patient may require more oxytocin.

**FACILITATOR’S GUIDE: *DURING THE SEMINAR***

1. At the beginning of the seminar, divide the students into small groups of 3-5 students.
2. Instruct groups to discuss the clinical vignettes and answer the pre-seminar quiz questions/discussion questions among themselves, consulting you when needed. This time of discussion should take approximately 40 minutes.
   1. As the facilitator, float from group to group answering any questions they may have and prompting responses to discussion questions with the topics listed on your answer sheet.
3. Then, instruct groups to discuss their results on the IAT and how bias may affect patient care for the next 15 minutes.
4. During the last 5-10 minutes of the seminar, distribute the post-seminar quizzes and feedback surveys among the students and advise them to take it individually. This will serve as a self-assessment of what they learned from the seminar.

**Total time:** (~30 min. advanced preparation from students) + 60-min. seminar = 90 min. learning experience

**OBESITY CBL FACILITATOR VERSION: DISCUSSION QUESTIONS**

**CASE #1: Patient is a 25-year-old G0 with Type 2 Diabetes Mellitus, Chronic Hypertension and BMI 48 presenting for preconception counseling. She is currently taking metformin and propranolol.**

1. What are some of the effects of obesity on fertility?

- Obesity is associated with decreased fertility due to oligo-ovulation and anovulation

- Obese patients are less likely to respond to ovulation induction, even with higher doses of gonadotropins

2. What are some of the maternal effects of obesity on pregnancy?

- Increased risk of gestational diabetes, preeclampsia, cesarean delivery, and infectious morbidity

- Operative morbidity increases due to difficulty of establishment and recovery from anesthesia, prolonged operating times, increased blood loss, and thromboembolism

- Less likely to have a successful vaginal birth after cesarean delivery

- Higher incidence of preterm birth

- Less likely to have spontaneous preterm labor

- More likely to be admitted earlier in labor, need labor induction, need increased doses of oxytocin, and have longer labor.

3. What are some fetal and neonatal effects of obesity in pregnancy?

- Increased risk of congenital anomalies, growth abnormalities, miscarriage, and stillbirth

- Birth defects related to neural tube, cardiac systems, and facial clefting

- Impaired visualization of ultrasound images which can compromise prenatal diagnoses of congenital anomalies

- Increased associated with subsequent childhood obesity

- Does not compromise fetal weight estimations

4. What are some weight-loss promoting medications that are suitable for this patient considering that she is trying to become pregnant?

- Many of the standard weight loss pharmacotherapy options are actually contraindicated in pregnancy, including phentermine and diethylpropion (norepinephrine-releasing agents), orlistat (pancreatic and gastric lipase inhibitor), and phentermine/topiramate (GABA receptor modulation plus norepinephrine-releasing agent).

- Use of antidiabetic medications that have additional actions to promote weight loss (such as glucagon-like peptide-1 [GLP-1] analogs or sodium-glucose-linked transporter-2 [SGLT-2] inhibitors), in addition to the first-line agent for T2DM and obesity, metformin, are excellent additions. A great example of a suitable drug is liraglutide.

- Should this patient need insulin, first-line recommendation is basal insulin plus the addition of either metformin, pramlintide, or a GLP-1 agonist in order to mitigate the potential weight gain from the insulin.

- Those with uncontrolled hypertension should not take medications that increase levels of norepinephrine (such as Phentermine, Diethylproprion or Buproprion).

5. What are the criteria for referral to bariatric surgery?

- BMI greater than 40

- BMI of 35 or more with other comorbities such as such as type II diabetes (T2DM), hypertension, sleep apnea and other respiratory disorders, non-alcoholic fatty liver disease, osteoarthritis, lipid abnormalities, gastrointestinal disorders, or heart disease

6. What are the two primary approaches to bariatric surgery weight loss and what is an example of each?

- Restrictive: restrict the amount of food that enters the GI system, example: adjustable gastric banding

- Restrictive and malabsorptive: restrict the amount of food and decrease absorption, example: Roux-en-Y gastric bypass

7. What effect does bariatric surgery have on future fertility?

- Rapid weight loss improves conditions such as PCOS, anovulation, and irregular menses, which can increase fertility

- Potential for compromised absorption of COCs after bariatric surgery

- While bariatric surgery can be beneficial for fertility issues, it should not be considered a treatment for infertility

**CASE #2: Patient is a 29-year-old G2P2002 status post gastric banding who previously had a BMI of 31 presenting for annual exam. She is also interested in contraception.**

1. Why is contraceptive counseling important in bariatric surgery patients?

- Pregnancy rates after bariatric surgery are double the rate in the general adolescent population

- There is an increased risk of oral contraception failure with a significant malabsorption component.

- Recommendation to wait 12-24 months after bariatric surgery to conceive so that the fetus is not exposed to rapid weight loss environment

2. What contraceptive options are available for this patient? If her prior surgery was malabsorptive, how would that change her options?

- Had her procedure been malabsorptive, there is potential for compromised absorption of COCs after bariatric surgery

- Non-oral administration of hormonal contraception should be considered in these patients

- May need to test drug levels for medications in which a therapeutic drug level is critical in order to ensure a therapeutic effect

3. How do contraception recommendations differ before and after bariatric surgery?

- Prior to bariatric surgery, patients with BMI > 30 or BMI > 27 with comorbidities, oral contraceptives over injectable medications are recommended due to possible weight gain with injectables

- After bariatric surgery, COCs are no longer recommended due to possible malabsorption

4. What are some of the most common nutritional deficiencies following bariatric surgery?

- Following Roux-en-Y gastric bypass, the most common deficiencies are protein, iron, vitamin B12, folate, vitamin D, and calcium

5. How do you monitor nutritional status in a pregnant patient who has had bariatric surgery?

- It is recommended to do a broad evaluation for micronutrient deficiencies at the beginning of the pregnancy, specifically looking for protein, iron, vitamin B12, folate, vitamin D, and calcium deficiencies

- If there is a proven deficit, provide appropriate treatment. Start with oral supplementation, but may begin parenteral supplementation if levels do not improve

- If no deficit is noted, monitor blood count, iron, ferritin, calcium, and vitamin D levels every trimester

6. Do patients who have had bariatric surgery require higher levels of certain vitamins and nutrients?

- Currently not known if bariatric surgery patients require higher doses of folate

- Many bariatric patients do not continue to take the prescribed multivitamin long term, therefore many patients without preconception counseling may not have adequate supplement levels at the beginning of pregnancy

- Daily recommendation of protein intake is the same regardless of bariatric surgery status (60 g daily)

7. What are the current nutritional recommendations for patients who have had bariatric surgery who become pregnant?

- These patients should take a prenatal vitamin in addition to a multivitamin

- Supplemental dosages of vitamin A should be limited to 5000 international units a day to avoid birth defects

- Consultation with a nutritionist after conception may help patient adhere to dietary regimens

8. What considerations must be made for a patient with a restrictive surgical procedure (such as gastric banding)?

- Patients may experience decreased food intake, intolerance to certain foods, or both

- May need to participate in "active band management" where fluid from the gastric band is removed or lessened during pregnancy; this may aid in nausea and vomiting during the first trimester

- Early consultation with a bariatric surgeon is recommended for these patients

**CASE #3: Patient is a 38-year-old G3P2002 at 9w3d, with a history of Roux-en-Y procedure, presenting to initiate prenatal care.**

1. What is an important consideration for this patient who has undergone bariatric surgery?

- In studies of pregnancy after bariatric surgery, many patients are still obese, with a prevalence up to 80%

- The possibility of continued obesity after surgery is important to consider when interpreting studies and clinical care options

2. How will this patient’s pregnancy compare to her pregnancies prior to the Roux-en-Y procedure?

- Rate of hypertension is decreased after surgery

- The occurrence of pre-gestational diabetes is decreased

- Decreased rates of gestational diabetes and preeclampsia

3. What is this patient’s chance of developing gestational diabetes after bariatric surgery?

- Compared to the general population, bariatric patients are more likely to develop gestational diabetes

- Comparing pregnancies before and after surgery, patients are less likely to develop gestational diabetes

4. How does bariatric surgery effect the rates of cesarean section?

- When compared to the general population, bariatric patients are more likely to 1) have had a prior cesarean section and 2) give birth via cesarean section

- When compared to nonobese patients who have not undergone surgery, there are higher cesarean section delivery rates after bariatric surgery; however, when compared to obese and severely obese controls, there is no significant difference

- The increase in cesarean section following bariatric surgery may be attributed to previous cesarean section in these patients

5. What are some of the effects of surgery on fetal and infant morbidity and mortality?

- The number of congenital anomalies after bariatric surgery is not increased when compared to the general population

- Some reports have noted lower mean birth weights indicating more appropriately grown infants, fewer LGA infants, and more SGA infants

- Macrosomia is decreased after Roux-en-Y gastric bypass

6. What considerations must be made during the prenatal period for this patient?

- There may be a delay in diagnosis of bariatric-related complications such as anastomotic leaks, bowel obstructions, internal hernias, ventral hernia, band erosion, and band migration

- All GI problems (such as nausea, vomiting, and abdominal pain) must be thoroughly evaluated and may need the involvement of the bariatric surgeon

- Some case reports have noted late severe complications of surgery such as maternal intestinal obstruction and gastrointestinal hemorrhage

- Exploratory surgery during pregnancy may be required to treat these patients

7. What is dumping syndrome and how may this affect prenatal screening tests?

- It is related to the ingestion of refined sugars or high glycemic carbohydrates that the stomach rapidly empties into the small intestine; fluid shift results in small bowel distention

- Symptoms include abdominal cramps, bloating, nausea/vomiting, and diarrhea. Later symptoms include hyperinsulinemia and consequent hypoglycemia, resulting in tachycardia, palpitations, anxiety, and diaphoresis

- Patients with dumping syndrome may not tolerate the 50g glucose solution routinely used at 24-28 weeks of gestation to screen for gestational diabetes

- One proposed alternative method of screening is to use home glucose monitoring for approximately one week during the 24-28 weeks of gestation.

8. What considerations must be made during labor and delivery for this patient?

- Because many patients remain obese even after bariatric surgery, these patients may be admitted earlier in labor, need labor induction, require more oxytocin, and have longer labor.

- Cesarean section rates are higher after bariatric surgery, although bariatric surgery itself should not be considered an indication for cesarean section

**In your small groups, discuss the outcomes of your IAT:**

- What were your overall thoughts of the assessment?
- Were your results similar or different from what you had expected?
- Do you think this test is truly predictive of your future behaviors?
- In what other ways do you think implicit bias could be assessed in student learners or healthcare professionals?

**OBESITY CBL FACILITATOR VERSION: POST-SEMINAR QUIZ**

**CASE #1: Patient is a 25-year-old G0 with Type 2 Diabetes Mellitus, Chronic Hypertension and BMI 48 presenting for preconception counseling. She is currently taking metformin and propranolol.**

1. What are some of the effects of obesity on this patient’s fertility?

a. Obesity can cause anovulation and oligo-ovulation.

b. Obese patients are more likely to respond to ovulation induction when given higher doses of gonadotropins.

c. Due to changes in estrogen levels, fertility actually increases in obese patients.

d. Obese patient have the same risk of miscarriage as the general population.

2. Which of the following is a suitable medication change for this patient considering that she is trying to become pregnant?

a. She should add orlistat in order to help her lose weight.

b. In addition to continuing her metformin, she should add a sulfonylurea to her medications in order to better control her diabetes.

c. She should switch from propranolol to lisinopril due to its renal protection in diabetes.

d. She should add liraglutide to her medications due to its weight loss properties.

3. Which of the following patients meet criteria for bariatric surgery?

a. 32 yo G1P1 with a BMI of 32 with type II diabetes

b. 28 yo G2P2 with a BMI of 38 who is otherwise healthy

c. 40 yo G3P2 with a BMI of 41 with sleep apnea

d. 25 yo G0P0 with a BMI of 34 with hypertension

**CASE #2: Patient is a 29-year-old G2P2002 status post gastric banding who previously had a BMI of 31 presenting for annual exam. She is also interested in contraception.**

4. Why is contraceptive counseling important in this patient?

a. Pregnancy rates after bariatric surgery are the same compared to the adolescent population.

b. It is recommended that patients wait 6 months after bariatric surgery to conceive.

c. There is increased risk of oral contraception failure should the patient choose to use COCs.

d. The patient can only use a non-hormonal option, such as a copper IUD, for contraception use.

5. Of the following, which contraceptive option is best for this patient following surgery?

a. Progestin-only pills

b. Combined oral contraception

c. Nexplanon

d. Copper IUD

6. Should this patient become pregnant, which of the following is false regarding the considerations that must be made for her history of gastric banding?

a. Removing fluid from the gastric band may aid in reducing nausea/vomiting in T1.

b. She may experience decreased food intake or food intolerance.

c. Due to this being a restrictive procedure rather than a malabsorptive procedure, the patient is at less risk of nutritional deficiencies.

d. Consultation with a bariatric surgeon is recommended.

**CASE #3: Patient is a 38-year-old G3P2002 at 9w3d, with a history of Roux-en-Y procedure, presenting to initiate prenatal care.**

7. How will this patient’s pregnancy compare to her pregnancies prior to the Roux-en-Y procedure?

a. There is a decreased rate of cesarean section.

b. There is a decreased rate of gestational diabetes.

c. There is a decreased rate of preterm delivery.

d. There is a decreased rate of PPROM.

8. According to some case reports, what is a severe complication of bariatric surgery that has been noted in pregnancy?

a. Gastrointestinal hemorrhage

b. Infections

c. Fistula formations

d. Gastrointestinal ileus

9. Which of the following is not a consideration that needs to be made during this patient's pregnancy?

a. There may be a delay in bariatric-related complications.

b. All GI problems must be thoroughly evaluated.

c. Exploratory surgery during pregnancy is not recommended due to possible risks.

d. Late severe complications of surgery may occur in pregnancy.

10. The patient presents to you during her pregnancy with abdominal cramps, diarrhea, and nausea/vomiting. Given her history of bariatric surgery, what is the likely diagnosis and what prenatal screening test does this affect?

a. Bowel obstruction; glucose challenge test

b. Ileus; urinalysis

c. GI leak; urinalysis

d. Dumping syndrome; glucose challenge test
