# Supplementary material for: Management of Obesity During Pregnancy and Periconception: Case-Based Learning for OB/GYN Clerkships
Source: MedEdPORTAL. 2021 Mar 23;17:11129. doi: 10.15766/mep_2374-8265.11129 (PMC8015635; doi:10.15766/mep_2374-8265.11129)
Supplement: Supplementary file 1 — Project Implicit Introduction.docxAdvance Preparation Student Version.docxFacilitator Guide.docxPreseminar Quiz Student Version.docxDiscussion Questions Student Version.docxPostseminar Feedback Survey.docx [file mep_2374-8265.11129-s001.zip › D. Preseminar Quiz Student Version.docx]

**OBESITY IN PREGNANCY CBL: PRE-SEMINAR QUIZ STUDENT VERSION**

**CASE #1: Patient is a 25-year-old G0 with Type 2 Diabetes Mellitus, Chronic Hypertension and BMI 48 presenting for preconception counseling. She is currently taking metformin and propranolol.**

1. Should this patient become pregnant, which of the following is false in regard to the maternal effects of obesity on her pregnancy?

a. This patient is at increased risk of gestational diabetes and preeclampsia.

b. This patient is more likely to have a spontaneous preterm labor.

c. This patient is more likely to have a cesarean delivery.

d. This patient is more likely to have a longer labor.

2. What should you tell this patient about the fetal and neonatal effects of obesity in pregnancy?

a. Being obese compromises fetal weight estimations.

b. Prenatal congenital anomalies are more likely to be diagnosed via ultrasound visualization in these patients.

c. There is a decreased risk of stillbirth in these patients.

d. There is an increased chance of birth defects relating to cardiac systems and facial clefting.

3. Should this patient undergo bariatric surgery, what effect will that have on her future fertility?

a. Rapid weight loss following surgery causes hormonal changes that decrease fertility.

b. There is the potential for compromised absorption of COCs following surgery.

c. Rapid weight loss can cause irregular menses following surgery.

d. She should wait at least 6 months after surgery to conceive so that the fetus is not exposed to rapid maternal weight loss.

**CASE #2: Patient is a 29-year-old G2P2002 status post gastric banding who previously had a BMI of 31 presenting for annual exam. She is also interested in contraception.**

4. Prior to her surgery, which of the following contraception options would be contraindicated in this patient?

a. Nexplanon

b. COCs

c. Depo shot

d. Levonorgestrel IUD

5. Which of the following is the least likely nutritional deficiency following bariatric surgery?

a. Iron

b. Folate

c. Vitamin B6

d. Calcium

6. Should this patient become pregnant; how will you manage her possible nutritional deficiencies?

a. Do a broad evaluation for micronutrient deficiencies starting in T2.

b. Get a CBC and micronutrient levels every trimester.

c. Start with parenteral supplementation if a specific nutritional deficit is noted.

d. Prescribe higher doses of folate due to her history of bariatric surgery.

**CASE #3: Patient is a 38-year-old G3P2002 at 9w3d, with a history of Roux-en-Y procedure, presenting to initiate prenatal care.**

7. What is an important consideration for this patient who has undergone bariatric surgery?

a. This patient has an increased risk of hypertension after surgery.

b. This patient is less likely to develop gestational diabetes when compared to the general public.

c. This patient has an increased risk of preeclampsia following surgery.

d. This patient is likely to still be obese, and this must be considered when interpreting studies and clinical care options.

8. Which of the following is false regarding the effect of bariatric surgery on the rates of cesarean section?

a. When compared to obese controls, there is no significant difference in rate of cesarean section.

b. When compared to the general population, bariatric patients are more likely to have had a prior cesarean section.

c. When compared to severely obese controls, there are lower rates of cesarean section delivery rates after bariatric surgery.

d. When compared to nonobese patients who have not undergone surgery, there are higher cesarean section delivery rates after bariatric surgery.

9. Which of the following is an effect of previous bariatric surgery on the fetus?

a. The fetus is at an increased risk of a congenital anomaly following bariatric surgery.

b. The rate of fetal macrosomia is the same compared to pre-bariatric surgery rates.

c. The baby is less likely to be large for gestational age compared to pre-bariatric surgery rates.

d. The baby is at an increased risk of perinatal death.

10. Which of the following is false regarding labor and delivery for this patient?

a. This patient may have a shorter labor time.

b. This patient may need labor induction.

c. This patient may be admitted earlier in labor.

d. This patient may require more oxytocin.
