# Supplementary material for: Management of Obesity During Pregnancy and Periconception: Case-Based Learning for OB/GYN Clerkships
Source: MedEdPORTAL. 2021 Mar 23;17:11129. doi: 10.15766/mep_2374-8265.11129 (PMC8015635; doi:10.15766/mep_2374-8265.11129)
Supplement: Supplementary file 1 — Project Implicit Introduction.docxAdvance Preparation Student Version.docxFacilitator Guide.docxPreseminar Quiz Student Version.docxDiscussion Questions Student Version.docxPostseminar Feedback Survey.docx [file mep_2374-8265.11129-s001.zip › E. Discussion Questions Student Version.docx]

**OBESITY IN PREGNANCY CBL: DISCUSSION QUESTIONS STUDENT VERSION**

**CASE #1: Patient is a 25-year-old G0 with Type 2 Diabetes Mellitus, Chronic Hypertension and BMI 48 presenting for preconception counseling. She is currently taking metformin and propranolol.**

1. What are some of the effects of obesity on fertility?

2. What are some of the maternal effects of obesity on pregnancy?

3. What are some fetal and neonatal effects of obesity in pregnancy?

4. What are some weight-loss promoting medications that are suitable for this patient considering that she is trying to become pregnant?

5. What are the criteria for referral to bariatric surgery?

6. What are the two primary approaches to bariatric surgery weight loss and what is an example of each?

7. What effect does bariatric surgery have on future fertility?

**CASE #2: Patient is a 29-year-old G2P2002 status post gastric banding who previously had a BMI of 31 presenting for annual exam. She is also interested in contraception.**

1. Why is contraceptive counseling important in bariatric surgery patients?

2. What contraceptive options are available for this patient? If her prior surgery was malabsorptive, how would that change her options?

3. How do contraception recommendations differ before and after bariatric surgery?

4. What are some of the most common nutritional deficiencies following bariatric surgery?

5. How do you monitor nutritional status in a pregnant patient who has had bariatric surgery?

6. Do patients who have had bariatric surgery require higher levels of certain vitamins and nutrients?

7. What are the current nutritional recommendations for patients who have had bariatric surgery who become pregnant?

8. What considerations must be made for a patient with a restrictive surgical procedure (such as gastric banding)?

**CASE #3: Patient is a 38-year-old G3P2002 at 9w3d, with a history of Roux-en-Y procedure, presenting to initiate prenatal care.**

1. What is an important consideration for this patient who has undergone bariatric surgery?

2. How will this patient’s pregnancy compare to her pregnancies prior to the Roux-en-Y procedure?

3. What is this patient’s chance of developing gestational diabetes after bariatric surgery?

4. How does bariatric surgery effect the rates of cesarean section?

5. What are some of the effects of surgery on fetal and infant morbidity and mortality?

6. What considerations must be made during the prenatal period for this patient?

7. What is dumping syndrome and how may this affect prenatal screening tests?

8. What considerations must be made during labor and delivery for this patient?

**In your small groups, discuss the outcomes of your IAT:**

- What were your overall thoughts of the assessment?
- Were your results similar or different from what you had expected?
- Do you think this test is truly predictive of your future behaviors?
- In what other ways do you think implicit bias could be assessed in student learners or healthcare professionals?
