# Supplementary material for: Management of Obesity During Pregnancy and Periconception: Case-Based Learning for OB/GYN Clerkships
Source: MedEdPORTAL. 2021 Mar 23;17:11129. doi: 10.15766/mep_2374-8265.11129 (PMC8015635; doi:10.15766/mep_2374-8265.11129)
Supplement: Supplementary file 1 — Project Implicit Introduction.docxAdvance Preparation Student Version.docxFacilitator Guide.docxPreseminar Quiz Student Version.docxDiscussion Questions Student Version.docxPostseminar Feedback Survey.docx [file mep_2374-8265.11129-s001.zip › F. Postseminar Feedback Survey.docx]

**OBESITY IN PREGNANCY CBL: POST-SEMINAR FEEDBACK SURVEY**

**Directions: Reflect on the seminar you just participated in by reading the following statements and circling the corresponding descriptive word/phrase (from “strongly agree” to “strongly disagree”) describing your level of agreement with it.**

1. Bias testing using the IAT was a meaningful experience for me.

Strongly agree Agree Neither agree nor disagree Disagree Strongly disagree

1. The discussion of cognitive bias was useful to me as a medical student.

Strongly agree Agree Neither agree nor disagree Disagree Strongly disagree

1. I will now be more intentional in considering my own bias when interacting with obese patients.

Strongly agree Agree Neither agree nor disagree Disagree Strongly disagree

1. The layout of the flipped classroom obesity CBL lesson was more engaging than a traditional CBL.

Strongly agree Agree Neither agree nor disagree Disagree Strongly disagree

1. The quizzes helped reinforce information taught in the obesity CBL.

Strongly agree Agree Neither agree nor disagree Disagree Strongly disagree

1. The CBL teaching method enabled me to be a more active learner than in the typical lecture-based style of teaching.

Strongly agree Agree Neither agree nor disagree Disagree Strongly disagree

1. Overall, I prefer the CBL method of teaching over the typical lecture-based style.

Strongly agree Agree Neither agree nor disagree Disagree Strongly disagree

1. Please leave any additional comments or concerns here:
