# Supplementary material for: Management of Obesity During Pregnancy and Periconception: Case-Based Learning for OB/GYN Clerkships
Source: MedEdPORTAL. 2021 Mar 23;17:11129. doi: 10.15766/mep_2374-8265.11129 (PMC8015635; doi:10.15766/mep_2374-8265.11129)
Supplement: Supplementary file 1 — Project Implicit Introduction.docxAdvance Preparation Student Version.docxFacilitator Guide.docxPreseminar Quiz Student Version.docxDiscussion Questions Student Version.docxPostseminar Feedback Survey.docx [file mep_2374-8265.11129-s001.zip › _Educational_Summary_Report_11129.pdf]

MedEdPORTAL® The AAMC Journal of Teaching and Learning Resources

Original Publication Open Access

Management of Obesity During Pregnancy and Periconception: Case-Based Learning for OB/GYN Clerkships

James Cook, MD*, Hannah L. Puckett, Jody E. Steinauer, MD, MAS

*Corresponding author: james.cook@uscmed.sc.edu

Abstract

Introduction: Medical students lack knowledge about the effects of bariatric surgery on pregnancy and medical management of obesity as it relates to reproductive health. Additionally, there is bias toward obese patients among clinicians and learners. Our goal is to improve knowledge and make students aware of the possibility of bias in their management of obese patients. **Methods:** We designed a flipped classroom learning experience focused on teaching students about the impacts and management of obesity and bariatric surgery on pregnancy and reproductive health. Before the seminar, students took the Implicit Attitude Test (IAT) and read two articles. During the 60-minute seminar, students worked in small groups discussing clinical vignettes, IAT results, and how bias affects patient care. One faculty preceptor oversaw the work and led discussions. We evaluated pilot seminars using Kirkpatrick levels 1 (reaction) and 2 (knowledge) outcomes. We measured change in knowledge after the seminar (using pre- and postseminar quizzes) and assessed students' feedback using a postseminar survey. **Results:** This module was piloted with one in-person group ($n = 9$) and one virtual group ($n = 11$) of third-year medical students. Students' knowledge improved (48% vs. 84% correct, $p < .001$), and they displayed statistically significant improvements in quiz scores postseminar. **Discussion:** Educators striving to teach learners about the management of obesity in pregnancy using evidence-based medicine should integrate this module into their medical student clerkship curricula.

Keywords
Bariatric Surgery, Obesity, Pregnancy, Reproductive Health, Women's Health, OB/GYN, Case-Based Learning, Clinical Teaching/Bedside Teaching, Flipped Classroom

Educational Objectives

By the end of this session, learners will be able to:

1. Explain the maternal and fetal effects of obesity on pregnancy.
2. Identify the indications for using pharmacotherapy versus bariatric surgery to manage obesity in a patient who desires future fertility.
3. Discuss the effects bariatric surgery has on future fertility and contraception.
4. Understand the impact bariatric surgery can have on future pregnancies.
5. Reflect on how implicit bias impacts the delivery of patient care to obese patients.

Citation:
Cook J, Puckett HL, Steinauer JE. Management of obesity during pregnancy and periconception: case-based learning for OB/GYN clerkships. *MedEdPORTAL*. 2021;17:11129.
https://doi.org/10.15766/mep_2374-8265.11129

Introduction

Obesity is defined as a body mass index (BMI) of 30 or more.[1,2] Over the past decade, the prevalence of obesity has been increasing worldwide, and now, more than 20% of the population of reproductive-aged individuals living in the U.S. are considered obese.[3] Treatment modalities for obesity include lifestyle changes, medical management, and even surgery, all of which have the potential to impact future pregnancies and reproductive health in general.

After extensively searching *MedEdPORTAL* using the terms *obesity, reproductive health, female, reproduction,* and *bariatric surgery,* we found no existing resources integrating the management of obesity alongside reproductive health. We further extended our search to other databases, including PubMed, Association of Professor of Gynecology and Obstetrics, and the Endocrine Society, and found that while plenty of publications regarding the interplay of obesity and reproductive health existed, none were formatted to serve as a teaching tool. Some educational and teaching resources consider medical

Copyright © 2021 Cook et al. This is an open-access publication distributed under the terms of the Creative Commons Attribution license.

management of obesity from the perspective of an internist,[4] but there remains a lack of readily available learning resources for medical students on the management of obesity as it relates to reproductive health and, specifically, the effects of bariatric surgery on pregnancy. As a result, our module was designed to build upon these resources by presenting the knowledge to guide this type of management from the viewpoint of an obstetrics provider.

Additionally, a negative bias toward obese patients among clinicians and learners has been documented in the literature. Yet such implicit biases are seldom talked about or reflected upon. Therefore, this module has also been designed to explore these implicit biases.[5,6] Overall, our goal was to build upon evidence-based approaches to obesity management[4] by teaching medical students how to reflect on innate biases so that they would be prepared to provide patient-centered care to pregnant patients.

Maternal obesity is associated with adverse outcomes for both mother and child. Pregnancies in obese patients are often complicated by placental abnormalities, displayed by higher rates of pre-eclampsia, as well as gestational diabetes, preterm birth, infections, cesarean delivery, and postpartum hemorrhage[3,7,8] to name just a few. Operative morbidity increases as a result of challenges to establishing and recovering from anesthesia, prolonged operating times, increased blood loss, and thromboembolism.[1,9] Obese mothers are more likely to be admitted earlier in labor, need labor induction, require increased doses of oxytocin, and endure a longer duration of labor.[1] The effects of maternal obesity on the fetus include early pregnancy loss, stillbirth, shoulder dystocia, growth abnormalities, and increased risk of congenital fetal malformations, including neural tube defects and facial clefting.[1,10] Excess weight on a pregnant patient impairs visualization of ultrasound images, which can compromise prenatal diagnoses of these congenital anomalies.[1] Maternal obesity not only poses long-term health risks for the mother but is also associated with subsequent childhood obesity, compromising the well-being of the next generation.[1,10] Therefore, the prevention and management of obesity among individuals of reproductive age should be viewed as a global health priority.[3,7,11]

When the combination of lifestyle changes, behavioral therapy, and pharmacotherapy fails to adequately manage a patient's weight, surgical interventions may be the next best option. Bariatric surgery is available for patients with a BMI of 40 or more or those with a BMI of 35 or more and other comorbidities.[1,12] The two types of bariatric surgery commonly performed today are the adjustable gastric banding and the Roux-en-Y gastric bypass. The number of bariatric surgeries performed annually increases drastically each year, with half of these patients being individuals of reproductive age.[1,13] The rapid weight loss following such a procedure often increases fertility through improvement of conditions such as irregular menses, anovulation, or polycystic ovarian syndrome. However, the procedure is not considered a treatment for infertility.

Numerous studies have suggested the potential for impaired absorption of oral contraceptives after malabsorptive bariatric surgery procedures. The American College of Obstetricians and Gynecologists (ACOG) reports decreased rates of hypertension, pregestational and gestational diabetes, pre-eclampsia, and average pregnancy weight gain after bariatric surgery.[1,12] Evidence also shows that the incidence of preterm premature rupture of membranes and cesarean deliveries increase after these procedures.[1] Bariatric surgery can also lead to nutritional deficiencies, which should be discussed with these patients, especially if they plan on becoming pregnant in the future. The most common nutritional deficiencies seen after gastric bypass surgery are of iron, calcium, folate, vitamin B12, vitamin D, and protein, and caring for these patients in pregnancy is complicated.[1] The current recommendation from ACOG is to conduct a broad evaluation of these micronutrient levels at the beginning of pregnancy in patients with a history of bariatric surgery. All pregnant patients with a history of bariatric surgery should take a prenatal vitamin in addition to a multivitamin.[1,12,13] Because bariatric surgery is so common and leads to complex care, it is critical that medical students learn how such procedures can impact reproductive health and future pregnancies.

Not only are obese patients biologically set up for obstacles when it comes to future fertility and pregnancy but the literature has also shown that implicit bias towards this patient population exists among providers and student learners.[5,6] Negative attitudes towards obese patients begin early in medical training, manifesting as both explicit and implicit biases. Unlike explicit biases, implicit biases exist outside of conscious awareness and are less subject to conscious manipulation secondary to social acceptability.[6] One study that examined the implicit weight bias among 4,732 medical students found that 74% exhibited bias.[14] The implicit weight bias amongst student learners is a problem that needs to be addressed as it affects clinical judgment and behavior.[15] One of the most widely used tests for identifying implicit biases is the Implicit Attitude Test (IAT).[15] While studies have not uniformly correlated IAT scores with clinician behaviors, some have found a correlation with disparities in patient experiences of care, one with observer ratings of care

Copyright © 2021 Cook et al. This is an open-access publication distributed under the terms of the Creative Commons Attribution license.

provided, and others with clinical decision-making in simulated vignettes.[15]

Clinicians are just as likely as nonclinicians to hold unconscious stereotypes or biases against certain groups of people regardless of how they are measured, and this may negatively affect the care provided. Luckily, evidence suggests that when people become sensitized to their unconscious biases, they can minimize cognitive errors,[16] such as those that can occur when treating obese patients. Within *MedEdPORTAL,* one current publication addresses this need for reflection in order to combat these unconscious biases, but it is directed specifically to health professional faculty search committees.[16] In other words, there is a lack of such resources directed towards medical student learning. As a result, our case-based learning (CBL) module contributes to the literature by drawing conscious attention to these biases and illustrating the importance of this self-reflection among medical students in order to change future actions.

The medical management of pregnant post-bariatric-surgery patients, as well as obesity in general with regard to reproductive health, focuses on preventing these associated complications and being able to identify and appropriately treat them when they arise.[8,10] Oftentimes, this task can become overwhelming given the lack of foundational knowledge among medical students on how to treat this specific subset of patients. We created this CBL module as an introduction to the medical and obstetric management of this patient population upon which future guidelines and treatment strategies can build. Of note, this module is geared toward more senior medical students, and it could also be applicable for residents caring for pregnant patients. Case-based teaching methods have been shown to be a more effective strategy in promoting critical thinking and decision-making skills among students when compared to conventional lecture-based methods of teaching.[17,18] The clinical vignettes provided in CBL modules give learners an opportunity to analyze real-life situations and formulate diagnoses and treatment plans in a safe environment. This encourages students to become active learners engaging in discussions with their colleagues. CBL utilizes a guided inquiry approach, which focuses on long-term retention and teaching in a more time-efficient manner compared to the open inquiry approach employed in problem-based learning.[17,19] Furthermore, evidence has shown that students have an enjoyable experience and express more positive feedback towards CBL styles than conventional styles of lecturing.[17,20] CBL affords students a more realistic, clinic-like learning experience that fuels the enthusiasm for learning and patient care physicians-in-training should embody.[17]

Methods

We designed a flipped classroom learning experience using prereading and clinical vignettes to teach third-year medical students about the impacts and management of obesity and bariatric surgery on pregnancy and reproductive health. In our pilot study, we instructed the student participants to prepare by reading a short excerpt about Harvard's Project Implicit (included here in Appendix A for ease of access and to ensure the durability of this CBL module and its supporting materials) and taking a short IAT (URL for testing platform listed in Appendices B and C). We also asked students to read two review articles: "ACOG Practice Bulletin No. 105: Bariatric Surgery and Pregnancy"[1] and "Pharmacological Management of Obesity: An Endocrine Society Clinical Practice Guideline."[21] This student preparation phase was estimated to take approximately 30 minutes and was described in Appendix B. Appendix C was designed as a comprehensive guide for the facilitator of the seminar and included the postseminar quiz and feedback survey, which were printed out for each student participant prior to the start of the seminar. Appendix C was also where the facilitator could find the highlighted answers to both the pre- and postseminar quizzes, as well as the discussion questions with their corresponding bulleted answers, which were intended to enable the facilitator to lead the seminar discussions. (For this publication, we removed a portion of the answer key to the discussion questions because it involved the use of medicine that is no longer FDA approved.) Appendix D contained the preseminar quiz.

During the first 5 minutes of the seminar, the students took the preseminar quiz (Appendix D). These quiz questions, as well as those found in the postseminar quiz (within Appendix C), were original questions formulated by the first author, who was board certified in both OB/GYN and obesity management. They were piloted in both our in-person and virtual learning cohorts. Of note, the preseminar quiz and postseminar quiz were formerly known as quiz A and quiz B, respectively. During our original study, we gave half of the participating student group quiz A and the other half quiz B as their preseminar evaluations. We then swapped the quiz versions at the end of the seminar, giving quiz B to the students who had previously received quiz A and quiz A to the students who had previously received quiz B, in order to obtain a quantitative measurement of student knowledge postseminar. The goal of this methodology was to provide all students with 10 questions directly related to the CBL module

Copyright © 2021 Cook et al. This is an open-access publication distributed under the terms of the Creative Commons Attribution license.

material prior to the seminar's discussions and 10 different, but equally relevant, questions after conducting the seminar. This allowed us to assess improvement in students' knowledge base and collect reliable data while preventing students from being able to simply search for the answers to the pretest questions and use them on the posttest. However, for use in traditional teaching sessions, we renamed the quizzes as preseminar quiz (Appendix D) and postseminar quiz (within Appendix C), to be given to all participating students at the appropriate times, in order to simplify and further standardize the steps of our CBL module.

During the 60-minute, in-person seminar, students first worked in small groups to discuss clinical vignettes, answer questions, and design treatment plans for approximately 40 minutes (Appendix E). They then discussed their results on the IAT, if applicable, and how bias could affect patient care for the next 15 minutes. Faculty preceptors oversaw the work and led discussions. During the feedback session at the end, students took the postseminar quiz (contained within Appendix C) as a self-assessment tool to see how much they had improved from the preseminar quiz and to identify any remaining areas of weakness. They were also asked to complete an anonymous postseminar feedback survey that took approximately 5 minutes (Appendix F). Overall, the preseminar preparation phase took approximately 30 minutes, followed by the 60-minute, in-person seminar discussion, totaling a 90-minute CBL experience.

In the midst of the COVID-19 pandemic, the mode of delivery of this seminar changed from in-person learning to the virtual environment in order to prioritize the safety of our students and faculty. While this was not a planned aspect of our pilot study, it provided us with the opportunity to compare virtual to in-person learning and became a part of the evaluation of this educational module. The virtual platform we utilized was Zoom Video Communications, a free platform offering quality video, audio, and wireless screen-sharing performance across Windows, Mac, Linus, iOS, Android, Blackberry, Zoom Rooms, and H.323/SIP room systems.[22]

We evaluated the workshop on Kirkpatrick levels 1 (reaction to the workshop) and 2 (learning and attitudes).[23] For level 1, we asked participants to describe their agreement on a 5-point Likert scale (from *strongly agree* to *strongly disagree*) with seven statements about the values of specific seminar elements and invited them to add open-ended feedback (Appendix F). For level 2, we calculated the change in scores between the preseminar and postseminar quizzes to assess knowledge gained. We compared these with a chi-square test. To assess attitude change, we asked participants to describe their level of agreement with a statement about bias awareness: "I will now be more intentional in considering my own bias when interacting with obese patients." For the survey and quiz questions, we also compared responses from learners in the virtual session with those in the in-person seminars using chi-square tests.

Results

This CBL module was implemented in the obstetrics and gynecology clerkship curriculum at the University of South Carolina School of Medicine - Columbia and was piloted among 20 third-year medical students. Students were instructed to prepare for the discussion as described above, and the session was scheduled for 60 minutes of classroom time.

Using pre- and postseminar quizzes, with facts taken from the prereading and learning objectives, we measured basic knowledge about obesity and bariatric surgery and their impacts on pregnancy and reproductive health. These multiple-choice questions were formulated to align specifically with Educational Objectives 1-4 of this module, while the IAT and postseminar feedback survey addressed Educational Objective 5 on implicit bias. More specifically, in the preseminar quiz, questions 1 and 2 aligned with Educational Objective 1, questions 3 and 4 with Educational Objective 3, and questions 5-10 with Educational Objective 4. Within the postseminar quiz, question 1 corresponded to Educational Objective 1, questions 2 and 3 to Educational Objective 2, questions 4 and 5 to Educational Objective 3, and questions 6-10 to Educational Objective 4. Students' scores improved from the pre- to the postmodule quiz (48% vs. 84% correct, $p < .001$). A p value less than or equal to .05 indicated strong evidence against the null hypothesis, and therefore, the improvement in scores after the implementation of the module was statistically significant. The increase in knowledge did not vary by in-person or virtual module (36% vs. 35% average increase in score from pre- to postseminar quiz).

We also assessed students' reactions to the seminar's discussion regarding bias towards obese patients and their overall impression of the seminar during the postseminar feedback survey (Appendix F). Twenty students were asked to read the statements on the survey and select from the response options shown in the Table. None of the participating students strongly disagreed with the impacts and value of elements of the seminar.

At the end of the surveys, students had an opportunity to leave any comments regarding the CBL or quizzes. Responses included the following: "fun to interact and bounce ideas off of each other,"

Copyright © 2021 Cook et al. This is an open-access publication distributed under the terms of the Creative Commons Attribution license.

"awesome discussion and real-life examples," and "the before and after quizzes helped me learn."

The facilitator reported that this CBL module generated thought-provoking discussions and active learning among all students. Participating parties expressed increased confidence in treating obese and post-bariatric-surgery pregnant patients after completing the module. Overall, this CBL was well received among its participants and facilitator.

Discussion

In order to ensure that patients of all sizes receive appropriate, individualized care, medical students should learn about the prevention and management of obesity as it relates to reproductive health care; however, such training is currently lacking in medical education. Our CBL module helps close the gap in this area of medical education by providing a resource that both explores the interplay between obesity and reproductive health and delivers this information in an effective and engaging way. Although some educational and teaching resources exist regarding medical management of obesity alone,[4] our module builds upon them by implementing the fundamentals of obesity management in the setting of reproductive health care and pregnancy and including reflection about implicit bias and how it affects health care delivery.

We designed this resource as a CBL module to promote critical thinking and decision-making skills among students in a more clinic-like environment compared to the conventional lecture-based setting.[17-20] In our pilot implementation of the module with 20 third-year medical students, we found a 36% increase in average score from the pre- to postseminar quiz. Since each of the two quizzes was composed of 10 different questions, our results suggest that the participating students learned the material rather than merely searching for the answers to the pretest questions. Thus, at least in the short term, we demonstrated positive outcomes at the Kirkpatrick levels of reaction and learning.[23] According to our postseminar feedback surveys, the overwhelming majority of participants agreed that the discussion on bias was useful and that they would consider their own biases when interacting with obese patients in the future (Table). However, a significant portion of students appeared to be neutral or to disagree with the survey question asking if the bias testing using the IAT was a meaningful experience (Table). This aligns with the mixed reviews seen in the literature.[15] While we still recommend using the IAT as a way to introduce the idea of implicit bias to medical students, we invite future users to explore why this sizable minority did not find the IAT useful; alternatively, we welcome suggestions for potential replacement exercises to introduce implicit bias. Regardless, the self-reflection and interpersonal discussions carried out through this obesity CBL module helped to highlight the importance of acting intentionally to treat all patients with the same respect and compassion.

Since we incorporated this CBL module into our curriculum, students have expressed positive feedback. More than half of the students in both the virtual and in-person groups preferred

Table. Virtual Seminar ($n = 11$) Versus In-Person Seminar ($n = 9$) Participant Responses to Postseminar Feedback Survey

	Strongly Agree (%)		Agree (%)		Neither Agree nor Disagree (%)		Disagree (%)		Strongly Disagree (%)	
Survey Question	Virtual	In Person	Virtual	In Person	Virtual	In Person	Virtual	In Person	Virtual	In Person
1. Bias testing using the IAT was a meaningful experience for me.	9	33	55	33	18	33	18	0	0	0
2. The discussion of cognitive bias was useful to me as a medical student.	18	44	73	44	9	11	0	0	0	0
3. I will now be more intentional in considering my own bias when interacting with obese patients.	9	44	73	44	9	11	9	0	0	0
4. The layout of the flipped classroom obesity CBL lesson was more engaging than a traditional CBL.	18	33	45	56	36	11	0	0	0	0
5. The quizzes helped reinforce information taught in the obesity CBL.	9	11	73	67	9	22	9	0	0	0
6. The CBL teaching method enabled me to be a more active learner than in the typical lecture-based style of teaching.	9	0	82	44	9	33	0	0	0	0
7. Overall, I prefer the CBL method of teaching over the typical lecture-based style.	27	22	36	44	36	22	0	11	0	0

Abbreviations: CBL, case-based learning; IAT, Implicit Attitude Test.

Copyright © 2021 Cook et al. This is an open-access publication distributed under the terms of the Creative Commons Attribution license.

this method of teaching over the typical lecture-based methods (Table). Nevertheless, improvements can always be made and limitations considered. One limitation is the small sample size (11 for the virtual seminar and nine for the in-person seminar, making the total size of the study 20) of our pilot group findings. By carrying out more seminars and allowing more medical students to participate in our CBL module, we will be able to further evaluate it.[24] Another limitation is that the faculty facilitator of our seminars was board certified in obesity. This may limit generalizability as he likely had more knowledge on the topic than the traditional OB/GYN generalist would. Nevertheless, the inclusion of the facilitator guide (Appendix C) will hopefully prevent this discrepancy in expertise from serving as an obstacle to future facilitators. Additionally, the module was implemented in one school in the southeast, also possibly limiting generalizability. Therefore, the efficacy of this learning resource could be further evaluated after its introduction in additional regions of the country.

Our curricular evaluation had limitations. Because of the COVID-19 pandemic, our two cohorts had similar, but not the same, educational experiences. While this gave us the opportunity to compare the modes of facilitation, utilizing only one mode would have allowed us to implement a standardized methodology for all participants. Moreover, although the quiz questions were formulated with the use of expert opinion, they had not been previously used or assessed for validity, which limits our ability to conclude with certainty that participants' knowledge improved. Also, because the quizzes had uneven distribution of multiple-choice questions for each learning objective, we cannot be certain that each objective was met with certainty. Although we believe the quizzes gave us a reasonable estimate of knowledge acquisition and served as learning tools for the participants, we will add questions and map them to all learning objectives in future CBL modules.

We have successfully implemented this module in our clerkship and hope to inspire educators at other institutions to do the same. We also plan to build on this module with more advanced ones that can be used for OB/GYN and family medicine residents. For instance, future modules could include topics such as prenatal screening, preconception counseling, and contraception counseling for obese patients, as well as the complexity of nutritional deficiencies in patients who desire pregnancy after bariatric procedures. For now, we believe our module can help ensure all physicians graduate with knowledge of obese individuals' unique gynecologic and obstetric health care needs.

Appendices

A. Project Implicit Introduction.docx
B. Advance Preparation Student Version.docx
C. Facilitator Guide.docx
D. Preseminar Quiz Student Version.docx
E. Discussion Questions Student Version.docx
F. Postseminar Feedback Survey.docx

All appendices are peer reviewed as integral parts of the Original Publication.

James Cook, MD: Clinical Associate Professor, Department of Obstetrics and Gynecology, Prisma Health Midlands Affiliate; Assistant Dean of Clinical Learning, Office of Curricular Affairs, University of South Carolina School of Medicine - Columbia; ORCID: https://orcid.org/0000-0003-0765-1079

Hannah L. Puckett: Fourth-Year Medical Student, University of South Carolina School of Medicine - Columbia; ORCID: https://orcid.org/0000-0001-6830-4826

Jody E. Steinauer, MD, MAS: Associate Professor, Department of Obstetrics, Gynecology, and Reproductive Sciences, Zuckerberg San Francisco General and University of California, San Francisco, School of Medicine; ORCID: https://orcid.org/0000-0001-7714-0106

Disclosures
None to report.

Funding/Support
None to report.

Ethical Approval
Reported as not applicable.

References

1. ACOG Practice Bulletin no. 105: bariatric surgery and pregnancy. *Obstet Gynecol*. 2009;113(6):1405-1413. https://doi.org/10.1097/AOG.0b013e3181ac0544
2. Ogden CL, Carroll MD, Fryar CD, Flegal KM. *Prevalence of Obesity Among Adults and Youth: United States, 2011–2014*. National Center for Health Statistics; 2015. NCHS Data Brief 219. https://www.cdc.gov/nchs/data/databriefs/db219.pdf
3. Broughton DE, Moley KH. Obesity and female infertility: potential mediators of obesity's impact. *Fertil Steril*. 2017;107(4):840-847. https://doi.org/10.1016/j.fertnstert.2017.01.017
4. Pasarica M, Topping D. An evidence-based approach to teaching obesity management to medical students. *MedEdPORTAL*.

Copyright © 2021 Cook et al. This is an open-access publication distributed under the terms of the Creative Commons Attribution license.

2017;13:10662.
https://doi.org/10.15766/mep_2374-8265.10662

5. Geller G, Watkins PA. Addressing medical students' negative bias toward patients with obesity through ethics education. *AMA J Ethics*. 2018;20(10):E948-E959. https://doi.org/10.1001/amajethics.2018.948
6. Baker TK, Smith GS, Jacobs NN, et al. A deeper look at implicit weight bias in medical students. *Adv Health Sci Educ Theory Pract*. 2017;22(4):889-900. https://doi.org/10.1007/s10459-016-9718-1
7. Pasquali R, Patton L, Gambineri A. Obesity and infertility. *Curr Opin Endocrinol Diabetes Obes*. 2007;14(6):482-487. https://doi.org/10.1097/MED.0b013e3282f1d6cb
8. Ma RCW, Schmidt MI, Tam WH, McIntyre HD, Catalano PM. Clinical management of pregnancy in the obese mother: before conception, during pregnancy, and post partum. *Lancet Diabetes Endocrinol*. 2016;4(12):1037-1049. https://doi.org/10.1016/S2213-8587(16)30278-9
9. Catalano PM, Shankar K. Obesity and pregnancy: mechanisms of short term and long term adverse consequences for mother and child. *BMJ*. 2017;356:j1. https://doi.org/10.1136/bmj.j1
10. Poston L, Caleyachetty R, Cnattingius S, et al. Preconceptional and maternal obesity: epidemiology and health consequences. *Lancet Diabetes Endocrinol*. 2016;4(12):1025-1036. https://doi.org/10.1016/S2213-8587(16)30217-0
11. Brewer CJ, Balen AH. The adverse effects of obesity on conception and implantation. *Reproduction*. 2010;140(3):347-364. https://doi.org/10.1530/REP-09-0568
12. Kominiarek MA. Preparing for and managing a pregnancy after bariatric surgery. *Semin Perinatol*. 2011;35(6):356-361. https://doi.org/10.1053/j.semperi.2011.05.022
13. Maggard M, Li Z, Yermilov I, et al. *Bariatric Surgery in Women of Reproductive Age: Special Concerns for Pregnancy.* Agency for Healthcare Research and Quality; 2008. Evidence Report/ Technology Assessment 169. https://archive.ahrq.gov/research/findings/evidence-based-reports/barirep-evidence-report.pdf
14. Phelan SM, Dovidio JF, Puhl RM, et al. Implicit and explicit weight bias in a national sample of 4,732 medical students: the Medical Student CHANGES Study. *Obesity*. 2014;22(4):1201-1208. https://doi.org/10.1002/oby.20687
15. FitzGerald C, Hurst S. Implicit bias in healthcare professionals: a systematic review. *BMC Med Ethics*. 2017;18:19. https://doi.org/10.1186/s12910-017-0179-8
16. Cahn PS. Recognizing and reckoning with unconscious bias: a workshop for health professions faculty search committees. *MedEdPORTAL*. 2017;13:10544. https://doi.org/10.15766/mep_2374-8265.10544
17. Tam J, Wadhwa A. A child with limb pain: a case-based learning module and teaching resource for pediatric infectious diseases. *MedEdPORTAL*. 2017;13:10605. https://doi.org/10.15766/mep_2374-8265.10605
18. Kim S, Phillips WR, Pinsky L, Brock D, Phillips K, Keary J. A conceptual framework for developing teaching cases: a review and synthesis of the literature across disciplines. *Med Educ*. 2006;40(9):867-876. https://doi.org/10.1111/j.1365-2929.2006.02544.x
19. Srinivasan M, Wilkes M, Stevenson F, Nguyen T, Slavin S. Comparing problem-based learning with case-based learning: effects of a major curricular shift at two institutions. *Acad Med*. 2007;82(1):74-82. https://doi.org/10.1097/01.ACM.0000249963.93776.aa
20. Thistlethwaite JE, Davies D, Ekeocha S, et al. The effectiveness of case-based learning in health professional education. A BEME systematic review: BEME Guide no. 23. *Med Teach*. 2012;34(6): e421-e444. https://doi.org/10.3109/0142159X.2012.680939
21. Apovian CM, Aronne LJ, Bessesen DH, et al. Pharmacological management of obesity: an Endocrine Society clinical practice guideline. *J Clin Endocrinol Metab*. 2015;100(2):342-362. https://doi.org/10.1210/jc.2014-3415
22. Valibia KGC. *Zoom*. University of California, Riverside. https://sitelicense.ucr.edu/files/zoom_for_instructors.pdf
23. Yardley S, Dornan T. Kirkpatrick's levels and education "evidence." *Med Educ*. 2012;46(1):97-106. https://doi.org/10.1111/j.1365-2923.2011.04076.x
24. Mascha EJ, Vetter TR. Significance, errors, power, and sample size: the blocking and tackling of statistics. *Anesth Analg*. 2018;126(2):691-698. https://doi.org/10.1213/ANE.0000000000002741

Received: June 27, 2020
Accepted: January 13, 2021
Published: March 23, 2021

Copyright © 2021 Cook et al. This is an open-access publication distributed under the terms of the Creative Commons Attribution license.
